# poolMC: Smart pooling of mRNA samples in microarray experiments

**DOI:** 10.1186/1471-2105-11-299

**Published:** 2010-06-02

**Authors:** Raghunandan M Kainkaryam, Angela Bruex, Anna C Gilbert, John Schiefelbein, Peter J Woolf

**Affiliations:** 1 Department of Chemical Engineering, University of Michigan, Ann Arbor MI 48109, USA; 2 Department of Molecular, Cellular, and Developmental Biology, University of Michigan Ann Arbor MI 48109, USA; 3 Department of Mathematics, University of Michigan, Ann Arbor MI 48109, USA; 4 Bioinformatics Program, University of Michigan, Ann Arbor MI 48109, USA

## Abstract

**Background:**

Typically, pooling of mRNA samples in microarray experiments implies mixing mRNA from several biological-replicate samples before hybridization onto a microarray chip. Here we describe an alternative smart pooling strategy in which different samples, not necessarily biological replicates, are pooled in an information theoretic efficient way. Further, each sample is tested on multiple chips, but always in pools made up of different samples. The end goal is to exploit the compressibility of microarray data to reduce the number of chips used and increase the robustness to noise in measurements.

**Results:**

A theoretical framework to perform smart pooling of mRNA samples in microarray experiments was established and the software implementation of the pooling and decoding algorithms was developed in MATLAB. A proof-of-concept smart pooled experiment was performed using validated biological samples on commercially available gene chips. Differential-expression analysis of the smart pooled data was performed and compared against the unpooled control experiment.

**Conclusions:**

The theoretical developments and experimental demonstration in this paper provide a useful starting point to investigate smart pooling of mRNA samples in microarray experiments. Although the smart pooled experiment did not compare favorably with the control, the experiment highlighted important conditions for the successful implementation of smart pooling - linearity of measurements, sparsity in data, and large experiment size.

## Background

Presently, pooling in microarray experiments refers to the act of mixing messenger RNA (mRNA) collected from several biological-replicate samples, before hybridization onto a microarray chip [1-6]. This form of pooling may be used to reduce biological variation, to lower costs by reducing the number of microarray chips used, and to overcome the problem of limited sample availability.

In this paper, we describe a different pooling strategy; a smart pooling strategy based on compression algorithms from digital communication theory. The smart pooling strategy is applied to a large number of diverse biological samples, not necessarily biological replicates, which are pooled and tested on several microarray chips based on a pre-specified pooling design. The mathematical properties of smart pooling designs ensure that each sample is tested on multiple chips, but always in pools made up of a different set of samples, such that, data from all the chips taken together capture the same information as the standard one-sample-one-chip approach. Because of the convolution step involved in testing pools of samples on multiple chips, the measurements made from the smart pooling strategy must be decoded to obtain the gene expression value of each gene in every sample. To save cost and to accurately transmit information across digital communication channels, where bandwidth is limited and the channel is noisy, a similar compression and recovery strategy is used. Similarly, smart pooling can achieve an overall savings by using fewer microarray chips than samples being tested. The built-in redundancy of testing each sample on multiple microarray chips can also provide robust expression measurements. The gains of compression and robustness from using smart pooling strategies motivated us to investigate this method. Smart pooling strategies have been used in other high-throughput biological applications such as blood testing [7], drug screening [8,9], protein-protein interaction mapping [10,11], genotyping [12,13], and others [14]. By using commercially available gene chips to implement smart pooling, our method also differs significantly from other attempts to design compressive sensing DNA microarrays that apply smart pooling at the level of probes [15].

The pooling and decoding strategy used in this paper we call poolMC - pooled microarray. poolMC is based on theoretical ideas from the field of compressive sensing, which has already demonstrated its utility in signal and image processing applications [16,17]. Compressive sensing takes advantage of the intrinsic compressibility of a data stream or set of experiments to produce experimental designs that require fewer experiments and provide greater robustness. Other experimental design strategies, derived from the field of group testing [14,18], have similar goals as compressive sensing but are not designed to obtain continuous-valued, quantitative measurements. A key requirement for any compression based experimental design is sparsity in the underlying data. The definition of sparsity in the context of gene expression data will be discussed in more detail in the following sub-section.

To test the poolMC strategy, we have carried out a small pooling experiment using validated biological samples on commercially available gene chips. In the context of this pooled experiment, we explain poolMC's pooling and decoding strategy. The results of this pooled experiment are then compared to the standard one-sample-one-chip method for the same set of samples. Finally, we propose the ideal setting for using poolMC and suggest its potential benefit for large microarray experiments.

### Sparsity

A key requirement for any smart pooling strategy is that the data must be sparse to allow compression. In this paper, the data describing a single gene's expression pattern across several biological samples is called its expression profile. A gene's expression profile is said to be sparse if, across several samples, there are only a small number of samples in which the gene's expression is significantly different from its median expression value in these samples. In this context "different" is ill defined, so for practical purposes, "different" can be viewed as meaning differential expression. If a gene is differentially expressed in only a small number of samples across many being tested, then it is possible to exploit this sparsity to reduce the number of microarray chips needed to obtain the gene expression profile by pooling or multiplexing samples.

**Figure 1:**
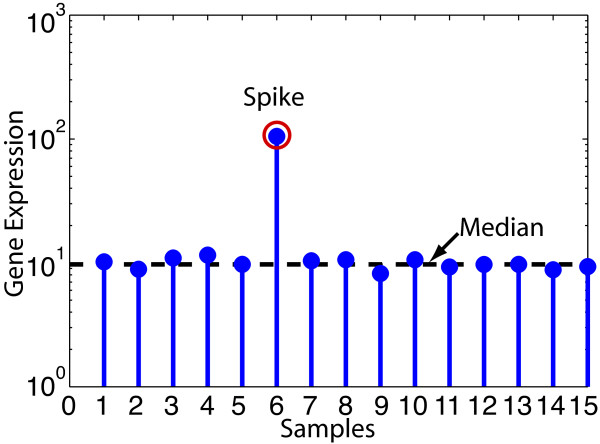
**Sparsity in a gene's expression profile**. Example of a gene showing only one spike (red circle) across 15 samples. The dotted line marks the median value for the samples.

Figure 1 shows an example of a gene with a sparse expression profile, because out of 15 samples the gene is differentially expressed in only one sample. By subtracting the median expression value from all samples only one sample, in this example, is left with a significant value while the rest of the expression values are close to zero. The significant value that is of interest is called a spike, as labeled in Figure 1. A spike can be a positive or negative deviation from the median depending on whether the gene was up or down regulated in a sample. Depending on the experiment there could be more or fewer spikes. For a particular experiment, different genes may display a wide variety in the number of spikes in their gene expression profile. However, when most, if not all, genes in an experiment have a sparse expression profile across a given set of samples, this sparsity can be exploited via smart pooling to compress the experiment into a smaller and more robust design. One way to achieve these savings while maintaining robustness is by pooling samples in microarray experiments.

Before describing the details of the pooling method, we first identify two cases of microarray experiments that could produce sparse gene expression profiles. A first example of sparsity could be mRNA samples obtained from similar cell samples, but with different, non-overlapping gene knockouts. This example case would bias the expression profiles toward sparsity because many of the genes would have no difference in expression (zero spike) across the various knockout samples. Those genes that do change would likely change only in one genetic background. A second example of a sparse gene expression study is in biomarker discovery where the samples, classified as treatment (or control), would have few genes differentially expressed in the samples within each classification, though they may show great variation when compared across each other.

The next sub-section describes the concept of smart pooling and how it exploits sparsity in gene expression profiles.

### Smart pooling

The central idea of smart pooling is to exploit sparsity in a gene's expression across several samples to obtain robust estimates of the gene's expression in all samples, while using fewer chips. This concept differs significantly from earlier microarray pooling attempts by not focusing on reducing the number of microarray chips used or increasing statistical power by estimating a single average expression value for each gene across multiple biological replicate samples. The goal of smart pooling is to recover the unique expression value of each gene in each of the samples being tested. To achieve this goal, samples are pooled in a systematic way across several chips while keeping the number of chips used lower than the number of samples tested. The pooling design ensures that each sample is tested on multiple chips, always in pools with different samples. The mathematical properties of the pooling design and decoding algorithm, discussed in more detail in the Methods section, guarantee that the expression of each gene in all samples is decoded accurately. For this strategy to succeed, two principle requirements need to be satisfied. First, as described in the previous sub-section, gene expression profiles need to be sparse. Second, the intensity of pooled measurements should map linearly to the contribution from each sample being pooled in the measurement.

**Figure 2:**
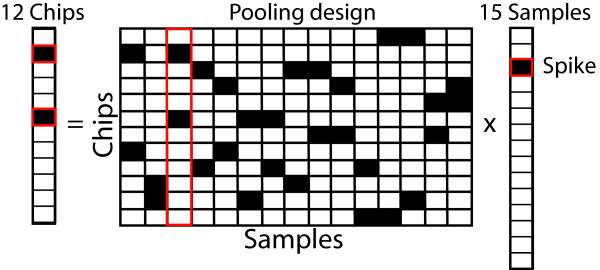
**Smart pooling process**. Schematic showing the pooling process and the utility of a sparse expression profile. The right column shows a gene's expression across 15 samples, with only 1 spike (highlighted dark square). Samples are mixed according to the pooling design in the middle. The columns of the pooling design represent the samples being pooled and the rows represent the microarray chips used to test them. A black square in the pooling design represents the presence of the sample (along that column) on the corresponding chip (along that row). The highlighted column in the pooling design shows the sample that corresponds to the spike. The left column shows the resulting measurements that contain only two significant values (dark squares), those coming from the sample with the spike.

If the data are sparse and mixing is additive, the pooling strategy can be reduced to a linear system of equations for each gene. As shown in Figure 2, the pooling design specifies the samples to be mixed and tested on microarray chips. However, in reducing the number of chips, we make fewer measurements than we have samples (e.g. 12 measurements of 15 samples in Figure 2). This mismatch between measurements and unknown variables (the gene's expression value in the samples) results in an underdetermined system of equations, which has an infinite number of solutions. However, by assuming that the underlying data are sparse, the number of possible solutions are reduced and the measurements contain enough information to uniquely solve the system of equations. Figure 2 illustrates this process. The right column shows a gene with only 1 spike (black square) in its expression profile, corresponding to sample number 3. When the samples are pooled according to a pre-specified pooling design shown in Figure 2 as a matrix, where a black square represents the presence of a sample (represented by a column) in a specific pool along that row, sample number 3 appears in pool numbers 2 and 6. The left column in Figure 2 shows the spike (black square) appearing in measurement numbers 2 and 6, as expected. Thus, the sparsity of an expression profile affects the number of useful measurements obtained by the pooled experiment thereby allowing the system of equations to be solved to obtain a unique solution. The redundancy attained through multiple measurements - two in this case - also provides a way to improve the robustness of the predictions. The theoretical basis for success of this pooling strategy is described in the next section.

## Results and Discussion

This section describes the pooling design and decoding algorithm that together form the poolMC smart pooling strategy. The details of the pooling design construction, the laboratory protocol for pooling, and the decoding of the results are described in the context of a small pooling experiment that was performed to test poolMC experimentally.

### Definitions

The current one-sample-one-chip microarray experiment is termed the monoplex. The pooled microarray experiment is called the multiplex. Because the data measured by the monoplex experiment are directly used, these data serve as both the monoplex measurements and the monoplex results. However, the multiplex experiment has two parts. The multiplex measurements refer to the data obtained from pooling samples on microarray chips, hence fewer measurements than the monoplex, and multiplex results refer to the data obtained from decoding the multiplex measurements. The multiplex results can be directly compared with the monoplex results. A synthetic mutliplex was performed using the monoplex results and multiplying them with the pooling design for each gene in the system. This produced synthetic measurements that were comparable to the multiplex measurement. The synthetic results, comparable to the monoplex and multiplex results, were obtained by decoding the synthetic measurements. The synthetic multiplex helped investigate the linearity assumption, by comparing multiplex and synthetic measurements, and the sparsity assumption by comparing the synthetic and monoplex results.

A proof of concept test was carried out using 15 mRNA samples obtained from the root epidermis of the plant Arabidopsis thaliana. These samples included 4 pairs of biological replicates, hence 8 samples, and 7 independent knockout samples. The monoplex measurements were performed at the usual concentration using 15 microarray chips. The multiplex measurements were obtained by mixing fractions of individual samples and using 12 microarray chips, employing the pooling design shown in Figure 2. The choice of this pooling design is explained in the next sub-section.

The mixing of fractions of individual samples was conducted at the mRNA level. Following isolation of mRNA from plant material (using the Qiagen RNeasy kit), the concentration of each sample was determined [19]. For the monoplex experiment 3-3.5 μg mRNA was used. For the multiplex the samples were pooled based on the pooling design described in the sub-section below, such that a total of 3.5 μg mRNA per chip was used. The cDNA was generated and labeled using the NuGen Ovation v2 and the NuGen Ovation FL kit. A total of 4 μg of the labeled cDNA were hybridized on the Affymetrix ATH1 Genechip.

The resultant mircoarray hybridization signal data corresponding to monoplex and multiplex measurements were preprocessed separately using RMA [20] and annotated using Brainarray custom CDF [21]. Each chip simultaneously measured the expression value of 21,505 genes. The microarray measurements obtained from the monoplex and the multiplex were normalized separately to ensure that the data were handled as though they were obtained from independent experiments, as typically expected. The preprocessed monoplex and multiplex experimental data are provided in Additional files 1 and 2 respectively.

### Theoretical details

Several pooling methods have been discussed in the literature [22,23]. The pooling design shown in Figure 2 and used in the experiment was based on an expander graph construction (defined in Definition 1 of the Methods section) used by Berinde et al. [24]. A detailed explanation of the pooling design is provided in Additional file 3 and MATLAB code for construction is provided in Additional file 4. For the experiment under consideration, the design tests 15 samples using 12 pooled tests and guarantees the recovery of 1 spike in a gene's expression profile.

The theoretical guarantee (defined in Theorem 1 of the Methods section and further explained in Additional file 3) is provided in the form of a bound on the error in decoded expression values. The amount of decoding error depends directly on the deviation of the actual expression values from the sparsity assumptions made by the pooling design. If the number of spikes in a gene's expression profile exceeds the design criterion, then the decoding error increases accordingly.

As described in the Background section, the problem of decoding reduces to solving an underdetermined system of equations, with more variables than measurements. Such a system would have an infinite number of solutions if an additional constraint of sparsity was not imposed. With such a constraint, however, the system has a unique solution that can be found by using a linear programming decoder [25]. The mathematical statement of the linear program is given in the Methods section. The MATLAB code for the decoder is provided in Additional file 5 and an illustrative example is provided in Additional file 6.

Further, measurement noise in experiments has a similar effect on decoding error as does deviation from sparsity. As the deviation from sparsity or experimental noise increases, the error in decoding gets worse. In the experiment under consideration, the pooling design guarantees the recovery of just 1 spike in the 15 samples being tested, when no experimental noise is present. Therefore, for genes with multiple spikes or single spikes with measurement noise the decoded result will not be exact. However, the mathematical guarantees are typically derived for worst-case scenarios. Smart pooling methods perform much better in practice, as demonstrated in the following sub-sections.

### Analysis procedure

The decoding procedure described in the previous sub-section was applied individually to each of the 21,505 genes in the system to obtain each gene's expression in all 15 samples.

Both the multiplex and synthetic pooled measurements were decoded and analyzed using the same procedure. The only source of noise in synthetic measurements is the noise resulting from monoplex measurements of the individual samples, and as such can be used to identify the sensitivity to experimental errors.

The success of poolMC was analyzed by directly comparing the monoplex and multiplex results. To test the linearity hypothesis, the multiplex measurements were compared with the synthetic measurements. To check if both datasets behaved similarly under standard microarray data analysis techniques, six differential expression analyses were performed between each of the 4 pairs of biological replicate samples present in the dataset for all three results - monoplex, multiplex, and synthetic. The overlap of significant genes obtained from the analysis in each of the six cases was used to evaluate the similarity of the monoplex to multiplex results.

### Experimental results

**Figure 3:**
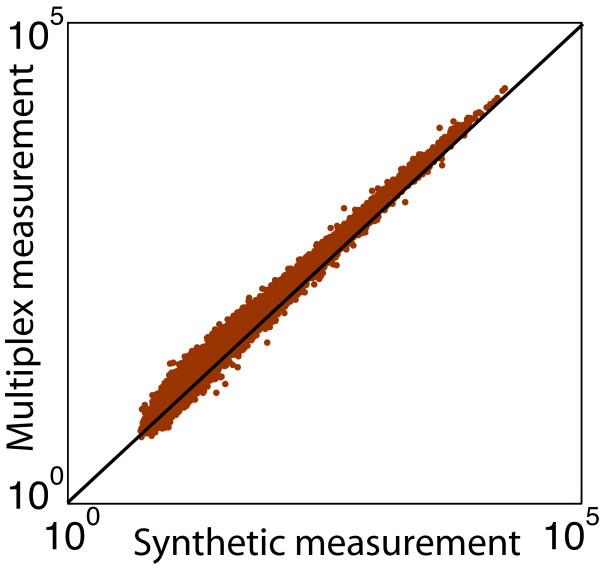
**Linearity of pooling process**. An example of the comparison between the expression data from a synthetic and a multiplex measurement showing data from all 21,505 genes for a single pooled chip

poolMC depends on two key assumptions - linearity and sparsity. To test the linearity assumption the multiplex measurement was compared with the synthetic measurement. Figure 3 shows an example of the alignment between synthetic and multiplex measurements, indicating that mixing samples produces measurements that are linearly additive. The same linear pattern can be observed for all 12 pooled chips, as shown in Additional file 3 (Supplementary Figure 6). In Figure 3, we observe a greater disagreement between the synthetic and multiplex data in the low expression range. This disagreement is likely due to measurement noise, because the synthetic measurements were simulated using the monoplex results, while the multiplex measurements were directly measured. As the expression level drops, the signal to noise ratio of the assay decays, generating discrepancies even between technical replicates. The slight deviation from the 45 degree line in Figure 3 is due to the monoplex, hence synthetic, and multiplex measurements being preprocessed separately.

Having confirmed the linearity assumption, the poolMC linear programming decoder was applied to both the synthetic and multiplex measurements. Overview figures for all samples are shown in Additional file 3 (Supplementary Figures 7 and 8). These results demonstrate that, overall, the synthetic case provides a better fit to the monoplex result. This better it is expected because the synthetic measurements have no measurement noise, relative to the monoplex. However, where the synthetic and multiplex results show large deviations from monoplex results, they do so in similar patterns, implying that the decoding error is due to deviation from the sparsity assumption of the design.

**Figure 4:**
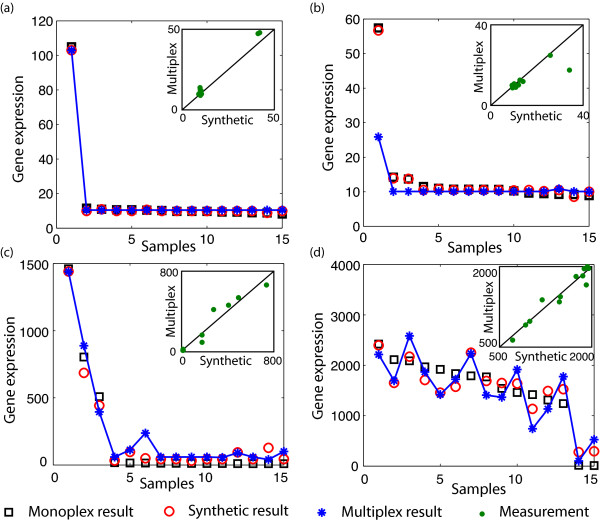
**Examples of poolMC results**. Four examples of decoding performance. (a) Low spike, low noise case, (b) Low spike, high noise case, (c) High spike, low noise case, and (d) High spike, high noise case. For each gene, expression profiles from monoplex result (black square), decoded synthetic result (red open circle), and decoded multiplex result (blue star with lines) are shown. Inset shows the alignment between synthetic and multiplex measurements (green dots) for the gene across the 12 pooled samples. Raw gene expression values are shown.

Next we examined the data at the individual gene level to determine how well the multiplexed results could recover the monoplexed results. Figure 4 shows four representative examples of individual genes, under different conditions of sparsity and measurement noise.

The four cases in Figure 4 are described below, numbered according to the figure.

a A gene with exactly 1 spike and close to no measurement noise (strong alignment between synthetic and multiplex measurements in inset) is decoded accurately, as guaranteed by the pooling design used.

b A gene with exactly 1 spike but with significant noise in a measurement (far right data point in inset is not aligned), is not decoded accurately.

c A gene with multiple spikes but low noise is decoded with moderate accuracy, even though the number of spikes exceeds the algorithmic guarantee.

d Gene with multiple spikes and low noise is not decoded accurately due to a larger deviation from the sparsity assumption than in (c).

Although the four examples are only a small sampling, the patterns shown in Figure 4 are representative of the properties of the decoding algorithm. The suggested experimental settings that will maximize the utility of smart pooling are discussed in the next section.

Given that the monoplex and multiplex results often produce the same pattern of expression, we next compared the lists of differentially expressed genes obtained from both lists. Because the 15 samples contain 4 pairs of replicate sample measurements (samples 2-4, 3-5, 7-8, and 12-15), differential expression analysis was performed between pairs of biological replicates using the Significance Analysis of Microarrays (SAM) method [26] to obtain lists of significantly expressed genes. The SAM analysis compares the expression data for two or more sample types (treatments or conditions) to identify genes that were differentially expressed among them. Further, it uses a permutation-based method to identify if the differential expression was significant. Hence the need for replicate measurements for each sample type. The SAM analysis was carried out independently within each dataset - monoplex, synthetic, and multiplex - and the resulting lists of differentially expressed genes were compared across the datasets (Table 1). Before carrying out the SAM analysis, 50% of the 21,505 genes were filtered out based on variance to increase statistical power [27].

**Table 1:** Evaluation of poolMC's performance

Sample pairs tested	# of significant genes at q = 10%			# Overlap with Monoplex	
	**Monoplex**	**Multiplex**	**Synthetic**	**Multiplex**	**Synthetic**

2-4 v. 3-5	761	43	13	27	13

2-4 v. 7-8	1545	3	5	3	5

2-4 v. 12-15	163	69	175	21	87

3-5 v. 7-8	1	0	0	0	0

3-5 v. 12-15	280	2	1	2	1

7-8 v. 12-15	26	1	1	0	1

Comparison of significant calls, obtained via a SAM analysis between biological replicate measurements from monoplex, multiplex, and synthetic datasets. The overlap of significant genes (at q-value = 10%) common to monoplex and each of multiplex and synthetic datasets for each biological replicate pair tested are shown.

In all six comparisons, the longest significant-gene lists were produced by the monoplex dataset. The multiplex and synthetic datasets identified fewer differentially expressed genes at the 10% q-value cut-off. As expected, the synthetic dataset shows more overlap with the monoplex than the multiplex does. This difference in overlap can be attributed to the decrease in noise in the synthetic dataset. The significant disparity in number of significant genes common to both monoplex and synthetic datasets can be attributed to the lack of sparsity in a large number of genes in the 15 samples chosen for the experiment, resulting in decoding errors.

## Conclusions

The aim of this work was to determine whether pooled experiments following a compressive sensing inspired design and decoder (poolMC) would be effective for gathering gene expression data. The approach described here is easy to implement with existing chips, as it only requires an intelligent mixing of samples. The analysis results indicate that gene expression measurements are sufficiently additive to be amenable to a linear decoder, and in some cases are sufficiently sparse to be compressed. When the experimental noise was sufficiently low and the expression profile was sparse, we have shown that poolMC can provide experimental compression.

**Figure 5:**
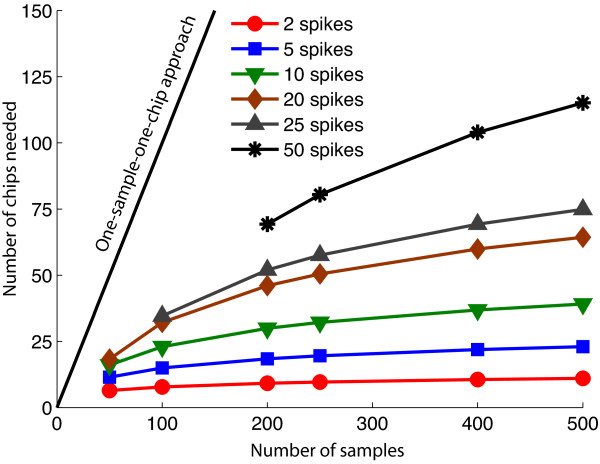
**Asymptotic performance of poolMC pooling design**. The approximate number of microarray chips needed based on number of samples used for the pooling experiment and the number of spikes expected in the samples.

However, the overall agreement between the lists of differentially expressed genes from the monoplex and multiplexed results did not show a strong overlap. This lack of overlap was due to three factors: (1) experiment size, (2) sparsity of gene expression, and (3) experimental noise. In this study, we carried out a small pilot study with only 12 multiplexed results compared to 15 monoplexed results. At this scale, multiplexing provides relatively few benefits in terms of both compression and robustness. As is shown in Figure 5, the compressive abilities of a poolMC type design increase significantly as the design is enlarged. The number of chips needed to identify *k *spikes in a experiment of size *n *is approximately *k log*(*n/k*) (see Additional file 3 for details). For example, a study with 100 samples would require approximately 23 chips, if only 1 in 10 samples showed differential expression. However, it should be noted that these calculations ignore measurement noise and should therefore be treated as a lower bound on the experiment size.

The second factor was the sparseness of the gene expression data. As is shown in Figure 5, the more spikes in a sample, the larger the required design. Gene expression data is not inherently sparse, but can be, depending on the particular samples chosen. In the experimental case used here, the data contain a large number of genes that do not change, corresponding to zero spike cases. These zero spike cases are, in general, accurately recovered from the multiplexing but of limited interest compared to cases that show differential expression. An ideal situation for using multiplexing to obtain gene expression data would be when testing a large number of samples to identify only a small number of unusual ones, such as when screening for a rare disease or mutation. Alternately, the decoding methods could incorporate information about the correlation structure of the genes to better exploit sparsity of the data. Finally, it should be noted that negative spikes could be more difficult to decode than positive spikes because negative spikes have a bounded magnitude that cannot go below zero. In contrast, up-regulated genes are essentially unbounded in their magnitude, and as such easier to decode.

The third factor is experimental noise. Gene expression profiling is a well-standardized method with relatively little noise, however this study shows that even the technical noise present in gene expression profiling can cause artifacts in multiplexed results. The experimental design can be modified to increase the robustness of the predictions to noise, but at the cost of adding additional experiments.

This small experiment suggests that pooling can be carried out successfully within the theoretical guarantees provided by poolMC. In practice, however, there are a number of strong requirements of experiment size, sparseness, and assay noise that need to be considered to make pooling work well for gene expression profiling. Figure 5 implies that for smart pooling to be successful, the number of samples used should be increased and the type of experiment should be chosen carefully to avoid a large number of spikes. Finally, it would be interesting to study the connections between the linear decoding procedure used in this paper and other ℓ_1 _regression methods such as Lasso [28].

## Methods

In this section, we provide the mathematical statements for the pooling design and decoding strategy that underlie poolMC. The practical implementation of these statements is provided in Additional file 3.

### Expander graph

An expander graph is a bipartite graph with high-connectivity. It connects a set of nodes on the left (*A*) to a set of nodes on the right (*B*) with a set of edges (*E*). The connections are such that each left node has a diverse set of neighbors on the right resulting in high-connectivity. The high-connectivity is useful for pooling as it ensures that no two samples are pooled together often. The graph can be represented as a binary matrix with the left nodes as columns, the right nodes as rows, and the edges as entries of the matrix; a matrix entry of value 1 represents the presence of an edge between the corresponding column and row nodes. This matrix is used as the pooling design. The mathematical definition of an expander graph, provided in [24], is as follows.

#### Definition 1

*A(k*, ϵ)-unbalanced expander is a bipartite simple graph *G-(A, B, E) *with left degree *d *such that for any *X *in *A *with |*X*| ≤ *k*, the set of neighbors *N*(*X*) of *X *has size |*N*(*X*)| ≥ (1 - ϵ) *d*|*X*|, where |·| represents the cardinality of a set.

In constructing such graphs for smart pooling, the goal is to make the right set size |*B*| (rows of the pooling matrix), *d *(number of times each sample is pooled), and ϵ (important for the theoretical guarantee of decoding) as small as possible. Details of their construction are provided Additional file 3 and MATLAB software implementation is provided in Additional file 4.

### Theoretical guarantee of decoding

poolMC provides the following decoding guarantee (from [24]):

#### Theorem 1

Let Φ be a *m *× *n *matrix of an unbalanced (2*k*, ϵ) expander. Consider any two vectors *x*, , such that  and . If *S *is the set of *k *largest (in magnitude) coefficients of *x*, then,

Here  is the decoded result of a gene expression profile measured by a pooling design Φ. If *x *is the "true" expression profile which contains *k *spikes, then the decoding error, represented as sum of absolute values (l1 norm) of the error in each entry of  with respect to *x*, is bounded by the l1 norm of the non-spikes in *x *(subtracting *x*_*S*_, which contains only the values corresponding to the *k *spikes, from x) and is scaled by a constant that depends on the properties of the pooling design Φ.

### Linear program decoder

The two conditions of  and  imposed on the decoded result  can be accomplished by finding a vector  using the following linear program:

poolMC uses the MATLAB-based software package l1-magic [25] to solve this linear program. This package can be downloaded from [25] and is required to execute the MATLAB code provided in Additional files 5 and 6.

## Authors' contributions

RMK framed the original problem, developed the algorithms and software, analyzed the data, and drafted the manuscript. AB and JS designed the experiment. AB performed the experiment. ACG and PJW participated in algorithm development and data analysis. All authors read and approved the final manuscript.

## Supplementary Material

Additional file 1 **Monoplex data**. Tab-delimited file containing the monoplex data. The file contains 21,505 rows corresponding to the genes and 15 columns of gene expression data (log-transformed) corresponding to the samples. Click here for file

Additional file 2 **Multiplex measurement**. Tab-delimited file containing the multiplex measurement. The file contains 21,505 rows corresponding to the genes and 12 columns of gene expression data (log-transformed) corresponding to the pooled samples. Click here for file

Additional file 3 **Supplementary materials**. An Adobe PDF file containing mathematical details and illustrative examples of the pooling and decoding strategies underlying poolMC. Also included are complete figures showing the comparison between monoplex, synthetic, and multiplex results for all samples used in the experiment. Click here for file

Additional file 4 **poolMC design**. The MATLAB code implementing the expander graph based pooling design. Click here for file

Additional file 5 **poolMC decoder**. The MATLAB code implementing the decoding procedure. Click here for file

Additional file 6 **Smart pooling example**. The MATLAB code illustrating poolMC's pooling and decoding procedure. Click here for file
