# Supplementary material for: poolMC: Smart pooling of mRNA samples in microarray experiments
Source: BMC Bioinformatics. 2010 Jun 2;11:299. doi: 10.1186/1471-2105-11-299 (PMC2900278; doi:10.1186/1471-2105-11-299)
Supplement: Additional file 3 — Supplementary materials. An Adobe PDF file containing mathematical details and illustrative examples of the pooling and decoding strategies underlying poolMC. Also included are complete figures showing the comparison between monoplex, synthetic, and multiplex results for all samples used in the experiment. [file 1471-2105-11-299-S3.PDF]

Supplementary materials – poolMC: Smart pooling of mRNA samples in microarray experiments

Raghunandan M Kainkaryam[1], Angela Bruex[2] , Anna C Gilbert[3] , John Schiefelbein[2] and Peter J Woolf*[1,4]

[1]Department of Chemical Engineering, University of Michigan, Ann Arbor MI 48109
[2]Department of Molecular, Cellular, and Developmental Biology, University of Michigan Ann Arbor MI 48109
[3]Department of Mathematics, University of Michigan, Ann Arbor MI 48109
[4]Bioinformatics Program, University of Michigan, Ann Arbor MI 48109

Email: Raghunandan M Kainkaryam - raghu@umich.edu; Angela Bruex - abruex@umich.edu; Anna C Gilbert - annacg@umich.edu; John Schiefelbein - schiefel@umich.edu; Peter J Woolf*- pwoolf@umich.edu;

*Corresponding author

Supplementary materials

Here we describe at greater length the mathematical basis and practical implementation of the poolMC smart pooling strategy. Also included are complete versions of the figures used in the paper to analyze the performance of poolMC in a pooled microarray experiment.

Pooling strategy

Several pooling methods have been discussed in the literature [1, 2] (Note: The reference numbers pertain to those cited in this document not the main paper). The design shown in Figure 2 of the paper and used in the experiment was based on an expander graph construction used by Berinde et. al. [3], which is defined as follows.

Definition 1

A (k, ϵ)-unbalanced expander is a bipartite simple graph $G - (A, B, E)$ with left degree d such that for any X in A with $|X| \leq k$, the set of neighbors $N(X)$ of X has size $|N(X)| \geq (1 - \epsilon)d|X|$, where $|\cdot|$ represents the cardinatlity of a set.

Supplementary Figure 1 illustrates this property of an expander graph in the context of the pooling design used in this paper. The pooling design tests 15 samples using 12 pooled chips. The expander graph shown

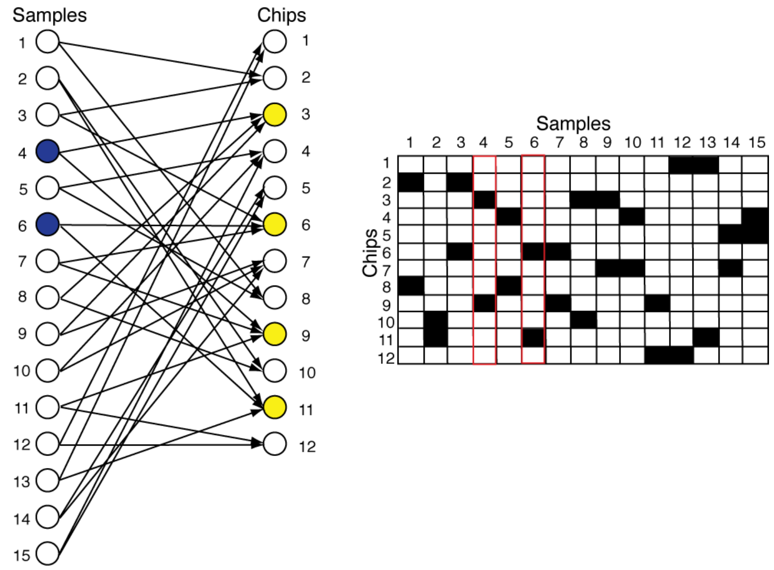

Supplementary Figure 1: Demonstration of expansion property of an expander graph. Two dark-colored nodes (samples) on the left of the graph expand to four different light-colored nodes (chips) on the right of the graph, thus providing 100% expansion ($\epsilon = 0$) for this particular pair of samples. The binary matrix on the right of the figure is the matrix representation of the expander graph on the left of the figure. Black squares in the matrix represent the presence of a sample (along the column) on a chip (along the row).

on the left of the figure can be represented as a binary matrix, shown on the right. The presence of a sample on a chip is represented by the value 1 (black square in Supplementary Figure 1). The left degree (d) of this graph is 2, that is, there are 2 edges emanating from each node on the left, which means each sample is tested on exactly 2 chips.

Definition 1 is illustrated with the example of samples 4 and 6, the left nodes shaded in blue in Supplementary Figure 1. The right neighbors for these two samples ($|X| = 2$ in Definition 1) are the chips numbered 3, 6, 9, and 11. Thus, $|N(X)|$ in Definition 1 is equal to 4. Therefore, ϵ in Definition 1 for this example is equal to 0. Similarly, the expansion coefficient ϵ can be calculated for other pairs of samples. For the design in Supplementary Figure 1, $\sim 77\%$ of all pairs have $\epsilon = 0$, the rest have $\epsilon = 0.25$. Therefore, for simplicity, the design used in this experiment can be considered an ($k = 2, \epsilon = 0$)-unbalanced expander graph, as defined in Definition 1. This simplification does not affect the demonstration of the theoretical guarantee of decoding. The utility of the expansion property of the pooling design is illustrated in the next section on the theoretical guarantee of decoding.

The pooling design is constructed by randomly placing d ones in each column of an $m \times n$ matrix; the rest

of the entries are zeros. This method guarantees a good expander graph, with high probability, when choice of d and m are made appropriately for a given n, k, and ϵ. Proposition 6 in [3], suggests that $d = O(\log(n/k)/\epsilon)$ and $m = O(k \log(n/k)/\epsilon^2)$. The MATLAB software implementation of the pooling design construction is provided as Additional File 4.

In practice, another constraint is imposed on the pooling design. The number of samples that can be mixed on each chip is limited, thereby placing a cap on the number of ones in each row of the pooling design matrix. However, since we need sparse matrices with good expansion properties, this cap is automatically satisfied, especially for large values of m and n. However, in the software implementation, we impose checks on the row weight of the pooling design and reject designs with high row weights. Further, instead of pooling each sample at full concentration, fractional concentrations of all samples are used in practice. The fractions are determined by row weights such that they sum up to a concentration equivalent to testing one sample, at full concentration, on each chip. Therefore, in practice, the pooling design is not a binary matrix, but has entries that are fractions summing up to one in each row. Finally, due to the small experiment size (12 chips for 15 samples), it was possible to exhaustively calculate the expansion property of the pooling design for low values of k (up to 2), thereby allowing us to choose a "good" design (where $\sim 77\%$ of all pairs of spikes had $\epsilon = 0$).

Decoding strategy

The poolMC decoder solves a linear program of the form:

$$\min ||\hat{x}||_1 \text{ subject to } ||\Phi\hat{x} - y||_2 \leq \delta \qquad \text{(P1)}$$

Here, Φ is the pooling design, y is the pooled measurement, and δ is a constraint relaxation parameter. poolMC uses the l1-magic software package [4] to solve the linear program. If the gene's expression profile is sparse, then Theorem 1 in the Methods section guarantees accurate decoding. Although, the guarantee in Theorem 1 is based on a non-relaxed version of P1.

As seen in Figure 1 of the paper, gene expression profiles are not sparse in the strictest sense. They can, however, be sparse when their deviation from the median value is considered. Therefore, poolMC's decoder operates on the median-subtracted gene expression. An approximate median value is arrived at from the median value of the pooled measurements. Since the pooling design used in practice preserves the magnitude of the gene's expression values (its rows sum up to 1), taking the median of measurements gives

a good approximation to the actual median value. The decoder then operates on the median-subtracted version of the measurements. The MATLAB software implementation of the poolMC decoder is provided in Additional File 5.

Theoretical guarantee of decoding

poolMC provides the following decoding guarantee (from [3]).

Theorem 1

Let Φ be a $m \times n$ matrix of an unbalanced $(2k, \epsilon)$ expander. Consider any two vectors $x, \hat{x}$, such that $\Phi x = \Phi \hat{x}$, and $||\hat{x}||_1 \leq ||x||_1$. If S is the set of k largest (in magnitude) coefficients of x, then,

$$||x - \hat{x}||_1 \leq C(\epsilon) \times ||x - x_S||_1$$

As shown in the previous section, the pooling design used in the experiment described in the paper is an (2,0)-unbalanced expander. Therefore, $k = 1$, in Theorem 1, which implies that genes with a single spike in their expression profile are guaranteed to be recovered with an error bounded by the right hand side expression. The error bound consists of two parts – the deviation from the one-spike assumption of the design and the scaling constant dependent on the design. The scaling constant is a function of the expansion coefficient ϵ of the expander graph, $C(\epsilon) = 2/(1 - 2\alpha(\epsilon))$ where $\alpha(\epsilon) = (2\epsilon)/(1 - 2\epsilon))$. Supplementary Figure 2 shows the relationship between these parameters. It would be advantageous to have a low value for ϵ as it would imply a low scaling factor. For $\epsilon = 0$, the scaling factor is 2. The effect of deviation from the sparsity assumption on the error guarantee of the decoding strategy is discussed next.

As shown in Figure 2 of the paper, the success of smart pooling depends on the sparsity of gene expression. Therefore, the theoretical guarantee for accurate recovery, Theorem 1, is stated in terms of the sparsity, or lack thereof, of the underlying gene expression. Conventionally, the mathematical arguments treat non-spikes as having a value close to zero. However, in the case of gene expression, the non-spikes have a value close to the median, as seen in Figure 1 of the paper. Therefore, a median-subtracted version of the gene expression profile is used in our analysis, a sample of which is shown in Supplementary Figure 3.

Theorem 1 can be restated as follows:

$$\%\text{Decoding} \quad \text{error} \leq \text{Constant} \times \%\text{Deviation} \quad \text{from} \quad \text{sparsity} \tag{1}$$

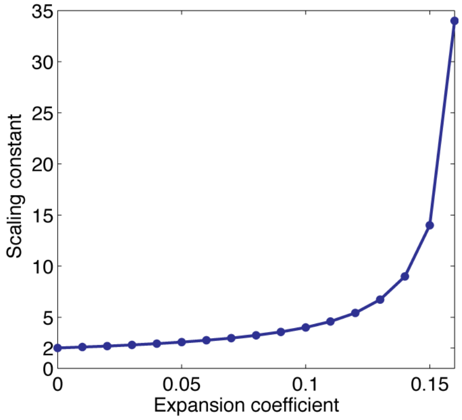

Supplementary Figure 2: Variation of the scaling constant in Theorem 1 as a function of the expansion property of the expander.

The decoding error refers to the total difference, across all samples, between decoded and actual (true) expression for a gene and is scaled to magnitude of the expression profile. According to equation 1, the percent decoding error will always be less than or equal to the percent deviation from the one-spike sparsity assumption made by the pooling scheme. The scaling constant depends on the pooling scheme used and is greater than or equal to 2, as described in the previous section. The guarantee provided by a smart pooling method, stated in equation 1, helps guide the choice of parameters for the pooling scheme design.

This result is illustrated using the example of the monoplex data for gene AT5G54740, shown in Supplementary Figure 3. For this gene, 90.41% of expression value is contained in a single sample. Therefore, we can use the pooling scheme described in the previous section, which was designed for a single spike.

Therefore, for the gene shown in Supplementary Figure 3, the deviation from the one-spike scenario is 100%-90.41%=9.59%. Therefore, the right-hand side (RHS) of equation 1 takes a value of 2 x 9.59% =19.18%.

A synthetic pooling experiment was performed using the data shown in Supplementary Figure 3, using the design shown in Supplementary Figure 1, in a noise-free setting. The decoded result of the synthetic pooling experiment is shown in Supplementary Figure 4. This example can be recreated by running the MATLAB code provided in Additional File 6.

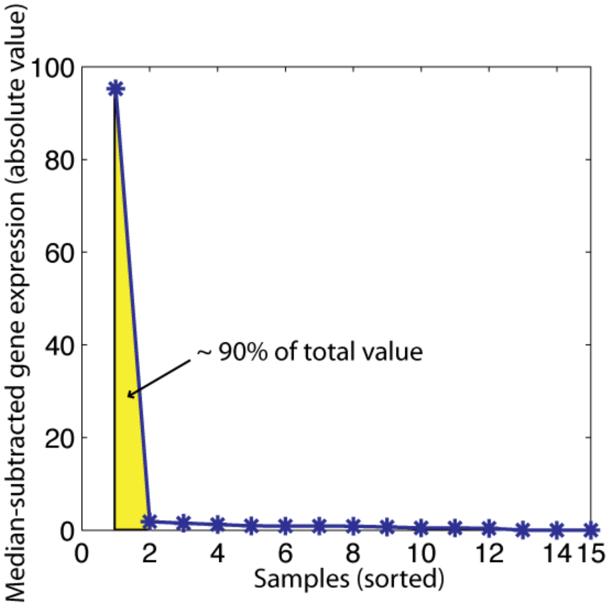

Supplementary Figure 3: Absolute value of median-subtracted gene expression for an example gene, sorted by magnitude.

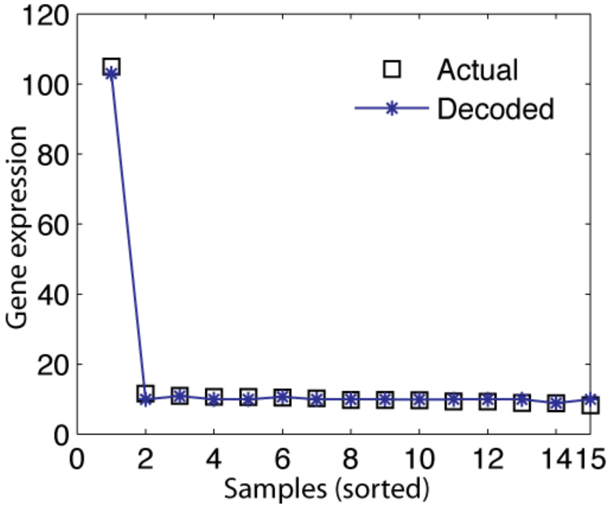

Supplementary Figure 4: Overlay of actual expression profile and decoded result of a synthetic pooling experiment for the gene shown in Supplementary Figure 3.

The percent decoding error, left-hand side (LHS) in equation 1, for the gene shown in Supplementary Figure 4 is equal to 9.39%. While the scaling constant times the percent deviation from sparsity, the right-hand side (RHS) of equation 1, was $2 \times 9.59\% = 19.18\%$. Therefore, the theoretical guarantee in equation 1 is satisfied.

The error guarantee in Theorem 1 is universal. Supplementary Figure 5 demonstrates this for the decoded results from the synthetic pooling experiment for all 21,505 genes in the system. As shown in the figure, the decoding error is always below the theoretical error bound guarantee. It also shows that the decoding error scales linearly with the deviation from sparsity.

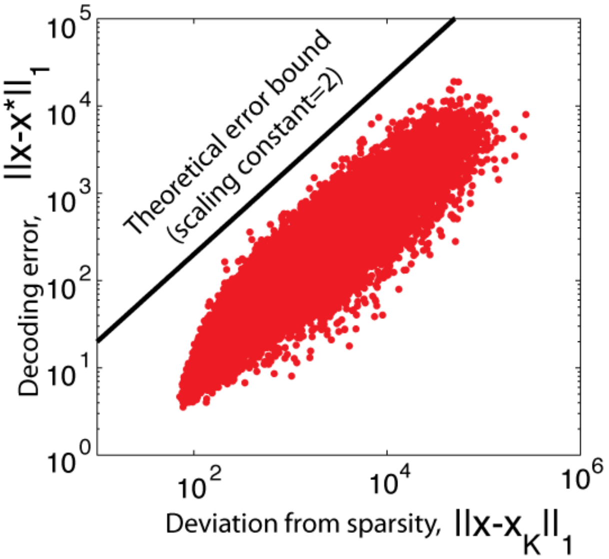

Supplementary Figure 5: A demonstration of Theorem 1 via a synthetic pooling experiment using the monoplex data, showing that the decoding error (y-axis) is always below the theoretical guarantee.

However, the conditions for the error bound in Theorem 1 are violated in practice in the following three ways:

1. In real systems there is noise in measurements. The effect of measurement noise is similar to deviation from sparsity in increasing the error bound on the decoded results.
2. As described in the pooling design section, the pooling design used in practice is not strictly a binary matrix (rescaled by a constant factor) as each row is rescaled so that it sums to 1. The effect of this deviation from the conditions under which Theorem 1 holds is not clear. Although, the synthetic and

experiments described in the paper were not affected by this change. For large enough pooling designs constructed randomly as in [3], the row weights cluster tightly about their mean value and our rescaling procedure produces a matrix which deviates only slightly from a truly binary matrix.

3. The poolMC decoder operates on the median-subtracted version of the measurements. However, the discrepancy in the estimation of the median adds to the decoding error. The decoding error due to median-approximation increases as the deviation from sparsity, hence the error in median estimation, increases.

Experimental results

Supplementary Figure 6 shows the complete comparisons between synthetic and multiplex measurements for all 12 pooled chips samples. As seen in the figure, the alignment between synthetic and multiplex measurements indicates that mixing samples produces measurements that are linearly additive. We observe a greater disagreement between the synthetic and multiplex data in the low expression range. This disagreement is likely due to measurement noise, because the synthetic measurements were simulated using the monoplex results, while the multiplex measurements were directly measured. As the expression level drops, the signal to noise ratio of the assay decays, generating discrepancies even between technical replicates. The slight deviation from the 45 degree line is due to the monoplex, hence synthetic, and multiplex measurements being pre-processed separately.

Having confirmed the linearity assumption, the poolMC linear programming decoder was applied to both the synthetic and multiplex measurements. Supplementary Figure 7 shows the comparison between monoplex and multiple results for all 15 samples, while Supplementary Figure 8 shows the comparison between monoplex and synthetic results for all 15 samples. These results demonstrate that overall the synthetic case provides a better fit to the monoplex result. This better fit is expected because of the synthetic measurements have less measurement noise than the multiplex measurements. However, where the two multiplex results show large deviations from monoplex results, they do so in similar patterns, implying that the decoding error is due to deviation from the sparsity assumption of the design.

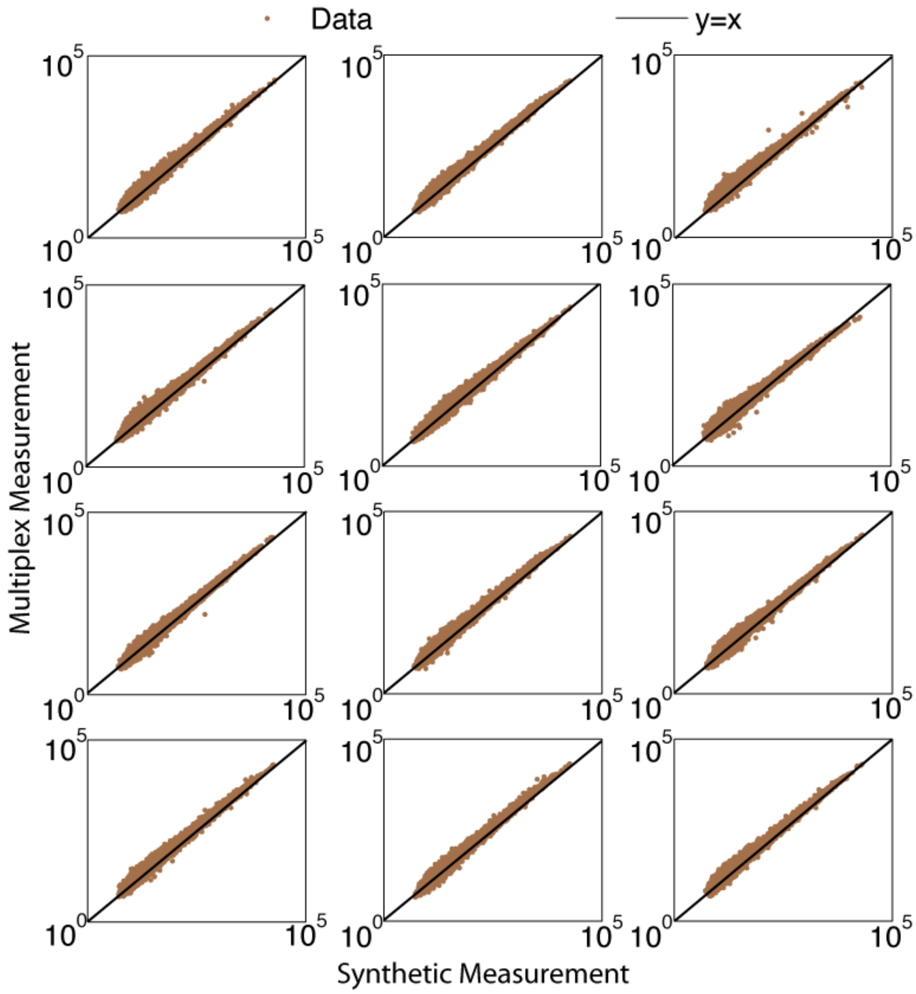

Supplementary Figure 6: Comparison between synthetic and multiplex measurement for all 12 pooled samples, showing all 21,505 genes.

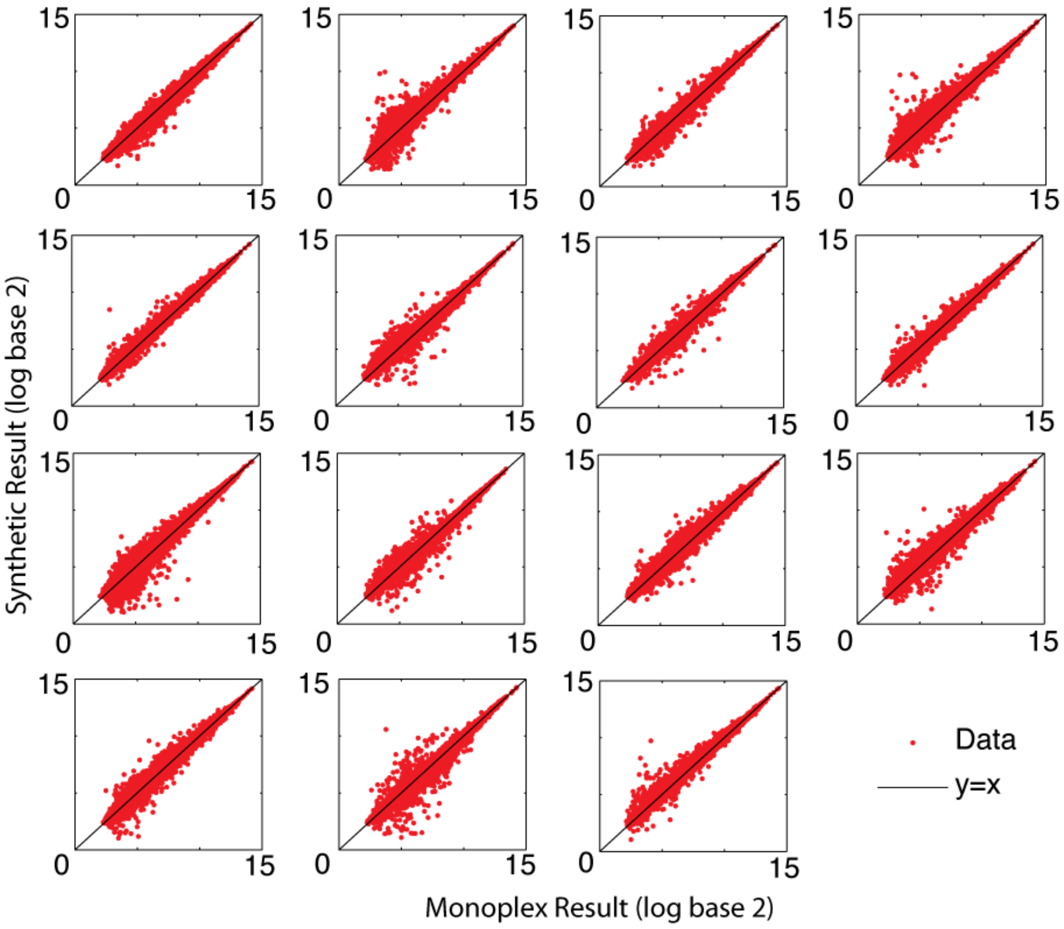

Supplementary Figure 7: Comparison between monoplex and synthetic pooling results for all 15 samples, showing all 21,505 genes.

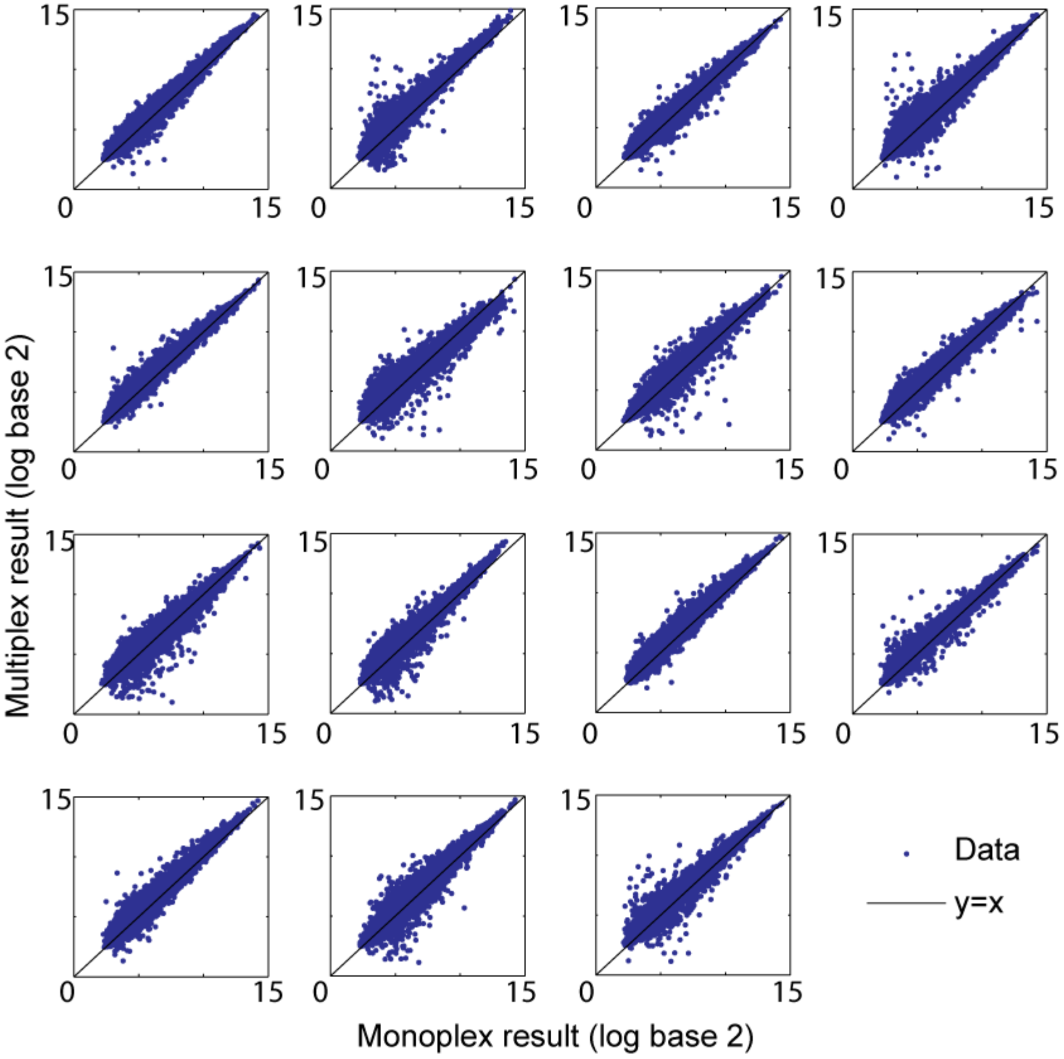

Supplementary Figure 8: Comparison between monoplex and multiplex results for all 15 samples, showing all 21,505 genes.

References

1. DeVore RA: **Deterministic constructions of compressed sensing matrices**. *J. Complex.* 2007, **23**(4-6):918–925, [http://dx.doi.org/10.1016/j.jco.2007.04.002].

2. Berinde R, Indyk P: **Sparse recovery using sparse random matrices**. *MIT-CSAIL Technical Report* 2008.

3. Berinde R, Gilbert A, Indyk P, Karloff H, Strauss M: **Combining geometry and combinatorics: A unified approach to sparse signal recovery**. In *46th Annual Allerton Conference on Communication, Control, and Computing* 2008:798–805.

4. Candès EJ, Romberg J: ℓ_1-*MAGIC: Recovery of Sparse Signals via Convex Programming* 2005. [Available at `http://www.acm.caltech.edu/l1magic`].
